# Asteraceae genome database: a comprehensive platform for Asteraceae genomics

**DOI:** 10.3389/fpls.2024.1445365

**Published:** 2024-08-19

**Authors:** Liang Wang, Hanting Yang, Guoqing Xu, Zhaoyu Liu, Fanbo Meng, LiangRui Shi, Xiongfeng Liu, Yixuan Zheng, Guichun Zhang, Xinyu Yang, Wei Chen, Chi Song, Boli Zhang

**Affiliations:** ^1^ State Key Laboratory of Modern Chinese Medicine, Tianjin University of Traditional Chinese Medicine, Tianjin, China; ^2^ China Resources Sanjiu Medical & Pharmaceutical Co., Ltd, Shenzhen, China; ^3^ Institute of Herbgenomics, Chengdu University of Traditional Chinese Medicine, Chengdu, China; ^4^ School of Basic Medical Sciences, Chengdu University of Traditional Chinese Medicine, Chengdu, China

**Keywords:** Asteraceae, genome, taxonomy, analysis tools, Asteraceae Genome Database (AGD)

## Abstract

Asteraceae, the largest family of angiosperms, has attracted widespread attention for its exceptional medicinal, horticultural, and ornamental value. However, researches on Asteraceae plants face challenges due to their intricate genetic background. With the continuous advancement of sequencing technology, a vast number of genomes and genetic resources from Asteraceae species have been accumulated. This has spurred a demand for comprehensive genomic analysis within this diverse plant group. To meet this need, we developed the Asteraceae Genomics Database (AGD; http://cbcb.cdutcm.edu.cn/AGD/). The AGD serves as a centralized and systematic resource, empowering researchers in various fields such as gene annotation, gene family analysis, evolutionary biology, and genetic breeding. AGD not only encompasses high-quality genomic sequences, and organelle genome data, but also provides a wide range of analytical tools, including BLAST, JBrowse, SSR Finder, HmmSearch, Heatmap, Primer3, PlantiSMASH, and CRISPRCasFinder. These tools enable users to conveniently query, analyze, and compare genomic information across various Asteraceae species. The establishment of AGD holds great significance in advancing Asteraceae genomics, promoting genetic breeding, and safeguarding biodiversity by providing researchers with a comprehensive and user-friendly genomics resource platform.

## 1 Introduction

Asteraceae, recognized as the largest family of angiosperms, is globally distributed and remarkably diverse. It encompasses over 1,600 genera and approximately 25,000 species (Shen et al., 2023), including notable members such as *Chrysanthemum morifolium*, *Artemisia caruifolia*, *Helianthus annuus*, and *Carthamus tinctorius* (Zhang and Elomaa, 2024). Chrysanthemum, a prominent perennial herbaceous plant within this family, holds a revered position among China’s top ten traditional flowers and is globally considered one of the four most preeminent cut flowers. Its geometrically regular inflorescences are visually appealing, contributing to the ornamental value of Asteraceae (Elomaa, 2019). In addition, the Asteraceae family holds important medical applications, significantly contributing to human health (Rolnik and Olas, 2021). Previous research has demonstrated that sesquiterpene lactones, naturally abundant in this family, possess anticancer potential (Li et al., 2020). Furthermore, Asteraceae can be employed as an *in vitro* antiplatelet agent and is utilized in diverse aspects of daily life, including cosmetics and food processing (Rolnik et al., 2022).

With the remarkable advancements in genome sequencing technology, substantial progress has been made in the genome research of various species, with much attention focused on Asteraceae in recent times. Particularly, *Helianthus annuus* (Badouin et al., 2017), *C. morifolium* (Song et al., 2023a), *C. nankingense* (Song et al., 2018), *Mikania micrantha* (Liu et al., 2020), *Artemisia annua* (Shen et al., 2018), and *Artemisia argyi* have all been extensively studied (Shen et al., 2018). Despite the numerous genomic studies conducted on various Asteraceae species, the genome sequences are distributed in different databases, lacking an integrated analysis platform and comprehensive databases that consolidate the vast amount of available information. Existing databases related to Asteraceae, including the Asteraceae genome size database (GSAD) (Garnatje et al., 2011), Asteraceae sequences database (Ventimiglia et al., 2023), burdock multi-omics database (Song et al., 2023b), and HeliantHOME (Bercovich et al., 2022). These databases do not systematically capture all the findings related to the Asteraceae genome. Such as GSAD only provides the function of querying the genome sizes of most Asteraceae species. Moreover, navigating through multiple platforms to obtain the required species data can be challenging and inconvenient. Therefore, developing a unique and comprehensive database, to provide researchers with a comprehensive platform for multi-omics research is crucial to consolidate and simplify access to Asteraceae genomic information.

In this work, we established the Asteraceae Genome Database (AGD), a comprehensive repository that integrates existing genome assembly and annotation data of representative Asteraceae species. We also regularly update the AGD to include new genomic data and research findings, ensure that AGD reflects the latest scientific advancements, and provide researchers with the most current information. We anticipate AGD evolving into a preeminent platform for the in-depth analyses of genomic data related to Asteraceae plants, streamlining access and interpretation of crucial information.

## 2 Database construction

### 2.1 Data retrieval

**Figure 1:**
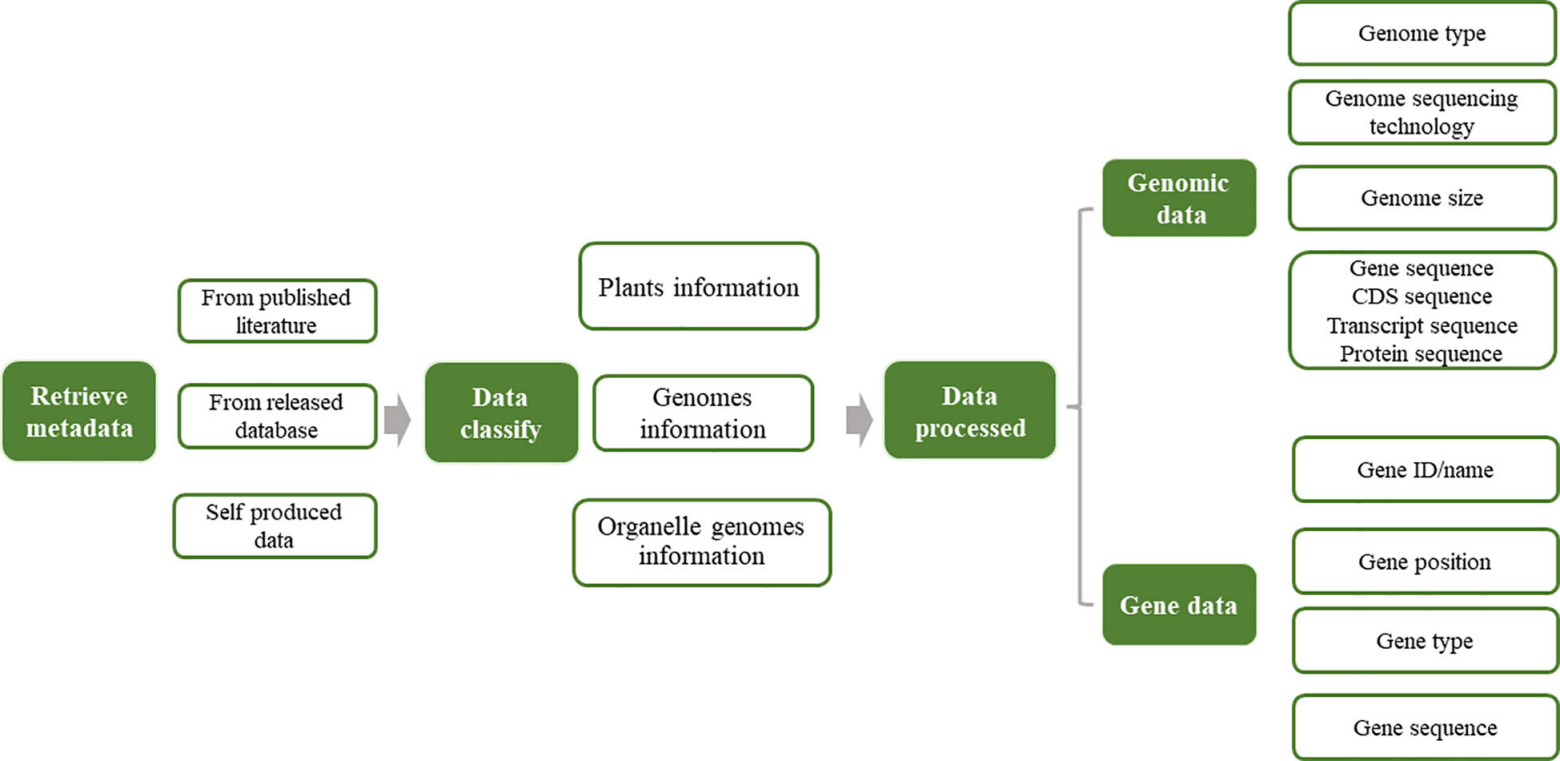
Analysis pipeline for data retrieval in AGD.

The complete omics data for Asteraceae were retrieved from various databases, including NCBI (National Center for Biotechnology Information, https://www.ncbi.nlm.nih.gov/), 1 K-MPGD (1 K Medicinal Plant Genome Database, http://www.herbgenome.com/) (Su et al., 2022), GPGD (Global Pharmacopoeia Genome Database, http://www.gpgenome.com) (Liao et al., 2022a), CNCB (China National Center for Bioinformation, https://www.cncb.ac.cn/?lang=en) (CNCB-NGDC Members and Partners, 2023), GWH (Genome Warehouse, https://ngdc.cncb.ac.cn/gwh) (Chen et al., 2021), Published Plant Genomes (https://www.plabipd.de/plant_genomes_pa.ep), and GERDH (Gene Expression Regulation Database of Horticultural plants, https://dphdatabase.com) (Cheng et al., 2023). We utilized the common and scientific nomenclature for species identification, for example, ‘Sunflowers’ and ‘*Helianthus annuus* L’, respectively, to facilitate a comprehensive retrieval of omics data. We expanded our keyword set to include the genus name and associated taxonomic designations to ensure a comprehensive search strategy. **Table 1** provides an overview of the extant genomic data available for the Asteraceae family. The AGD encompasses a diverse array of genomic data, including organelle and nuclear genomes. We employed the gffread tool (https://github.com/gpertea/gffread) to extract protein-coding, protein, and transcript sequences. These sequences were subsequently curated and integrated into our database. **Figure 1** presents the analysis pipeline employed by AGD.

**Table 1:** Species and genome data in Asteraceae.

Species	Accession number	Assembly Level	Genome size	References
*Arctium lappa*	JAKOEK000000000	Chromosome	1.73 Gb	(Fan et al., 2022)
*Carthamus tinctorius*	GWHBJIR00000000	Chromosome	1.17 Gb	(Chen et al., 2023a)
*Cynara cardunculus*	SUB874020	Chromosome	1,084 Mb	(Scaglione et al., 2016)
*Saussurea involucrata*	SAMN36288184	Chromosome	2452 Mb	(Sun et al., 2023)
*Silybum marianum *	JAWIMA000000000	Chromosome	694.4 Mb	(Kim et al., 2024)
*Ambrosia artemisiifolia*	PRJNA967341	Chromosome	1.13 Gb	(Laforest et al., 2024)
*Ambrosia trifida*	PRJNA967341	Chromosome	2.02 Gb	(Laforest et al., 2024)
*Artemisia argyi*	PRJCA010808	Chromosome	3.89 Gb	(Chen et al., 2023b)
*Helianthus annuus*	MNCJ02000000	Chromosome	3.6 Gb	(Badouin et al., 2017)
*Mikania micrantha*	SZYD00000000	Chromosome	1.8 Gb	(Liu et al., 2020)
*Lactuca sativa*	PRJCA007442	Chromosome	2.6 Gb	(Shen et al., 2023)
*Artemisia annua*	–	Chromosome	1.11 Gb	(Liao et al., 2022b)
*Erigeron breviscapus*	PRJNA525743	Chromosome	1.4 Gb	(He et al., 2021)
*Bidens hawaiensis*	SAMN18676211	Chromosome	6.67 Gb	(Bellinger et al., 2022)
*Artemisia tridentata*	SAMN24662005	Chromosome	4.2Gb	(Melton et al., 2022)
*Chrysanthemum indicum*	–	Chromosome	3.11Gb	(Deng et al., 2024)
*Chrysanthemum lavandulifolium*	JAHFWF000000000	Chromosome	2.60 Gb	(Wen et al., 2022)
*Chrysanthemum makinoi*	JP131333	Chromosome	3.1 Gb	(Van Lieshout et al., 2022)
*Chrysanthemum nankingense*	–	Chromosome	3.07 Gb	(Song et al., 2018)
*Chrysanthemum seticuspe*	GCA_019973895.1	Chromosome	3.05 Gb	(Nakano et al., 2021)
*Chrysanthemum morifolium*	PRJNA796762 PRJNA895586	Chromosome	8.15 Gb	(Song et al., 2023a)
*Conyza canadensis*	SUB535309	Chromosome	335 Mb	(Peng et al., 2014)
*Dittrichia graveolens*	PRJNA919087-8	Chromosome	835 Mb	(McEvoy et al., 2023)
*Glebionis coronaria*	JANFOE000000000	Chromosome	6.8 Gb	(Wang et al., 2022)
*Helianthus tuberosus*	PRJNA918503	Chromosome	21Gb	(Wang et al., 2024)
*Helichrysum umbraculigerum*	PRJEB52026	Chromosome	1.3 Gb	(Berman et al., 2023)
*Pluchea indica*	PRJCA004930	Chromosome	495.4 Mb	(He et al., 2022)
*Pulicaria dysenterica*	PRJEB50479	Chromosome	833.2Mb	(Christenhusz et al., 2023)
*Scalesia atractyloides*	PRJEB52418	Chromosome	3.2Gb	(Cerca et al., 2022)
*Smallanthus sonchifolius*	JAKNSE000000000	Chromosome	2.72 Gb	(Fan et al., 2022)
*Stevia rebaudiana*	PRJNA684944	Chromosome	1416 Mb	(Xu et al., 2021)
*Tagetes erecta*	–	Chromosome	707.21Mb	(Xin et al., 2023)
*Tanacetum cinerariifolium*	PRJDB8358	Chromosome	7.1Gb	(Yamashiro et al., 2019)
*Tanacetum coccineum*	PSUB016075	Chromosome	9.4 Gb	(Yamashiro et al., 2022)
*Cichorium endivia*	JAKOPN000000000	Chromosome	0.89Gb	(Zhang et al., 2022)
*Cichorium intybus*	JAKNSD000000000	Chromosome	1.28Gb	(Fan et al., 2022)
*Lactuca saligna*	PRJEB56287	Chromosome	2.27 Gb	(Shen et al., 2023)
*Lactuca virosa*	PRJEB50301	Chromosome	3.7 Gb	(Xiong et al., 2023)
*Taraxacum mongolicum*	PRJCA005187	Chromosome	790 Mb	(Lin et al., 2022)
*Taraxacum kok-saghyz*	PRJCA005187	Chromosome	1.1 Gb	(Lin et al., 2022)

### 2.2 Supplements to plant and genome information

Taxonomic resources and phenotypic images were obtained from iplant (https://www.iplant.cn/), Wikipedia (https://encyclopedia.thefreedictionary.com/), and Flora of China (http://flora.huh.harvard.edu/china/mss/intindex.htm). We documented the key details of each genomic publication, including the title, publication date, journal, and the unique PubMed identifier. We conducted a careful manual review of the associated academic articles for each genome to obtain information such as the genome size, assembly level, and the number of predicted genes. Moreover, we extracted the details of the pertinent annotation files.

### 2.3 Database implementation

The database is supported by Django (https://www.djangoproject.com/), uWSGI (https://uwsgi-docs-zh.readthedocs.io/zh-cn/latest/), and Nginx (https://nginx.org/en/). MySQL (https://www.mysql.com/) is used for the data management and organization of AGD. To provide a smooth and friendly user interface, bootstrap (v.4, https://v4.bootcss.com/), fontawesome (v.free-6.4.0, https://fontawesome.com/), and layUI, (https://layui.dev/docs/2/form/select.html#normal) were employed to improve the interface visual. The statistical results are displayed using bootstrap-table (https://getbootstrap.com/docs/4.0/content/tables/) and ECharts (https://echarts.apache.org/zh/index.html).

### 2.4 Analysis tools

Eight bioinformatics tools have been integrated into AGD, namely, BLAST (Camacho et al., 2009), JBrowse (Skinner et al., 2009), SSR Finder (Castelo et al., 2002), Heatmap (Verhaak et al., 2006), Primer3 (Rozen and Skaletsky, 2000), PlantiSMASH (Kautsar et al., 2017), CRISPRCasFIDER (Couvin et al., 2018), and HmmSearch (Rehmsmeier and Vingron, 2001). The BLAST service was constructed using the SequenceServer application, which serves as a robust front-end for BLAST. The AGD capabilities are enhanced by embedding JBrowse 2, a new version of the genome visualization tool (Diesh et al., 2023). The SSR web interface was developed to identify SSRs in user-submitted sequences, taking inspiration from the MISA page (https://webblast.ipk-gatersleben.de/misa/index.php?action=1). Protein domains are identified using the HmmSearch program within the HMMER (v.3.3.2) software suite. The Heatmap tool can provide the heat map determined from the expression profile data. Moreover, a PCR primer design tool is embedded into the system, allowing users to adopt the capabilities of Primer. PlantiSMASH is integrated to detect known secondary metabolic gene clusters present within chromosome-level genomes. The identification of CRISPR arrays and Cas proteins is facilitated by the tools provided within the AGD platform.

## 3 Results

### 3.1 Structure of AGD

**Figure 2:**
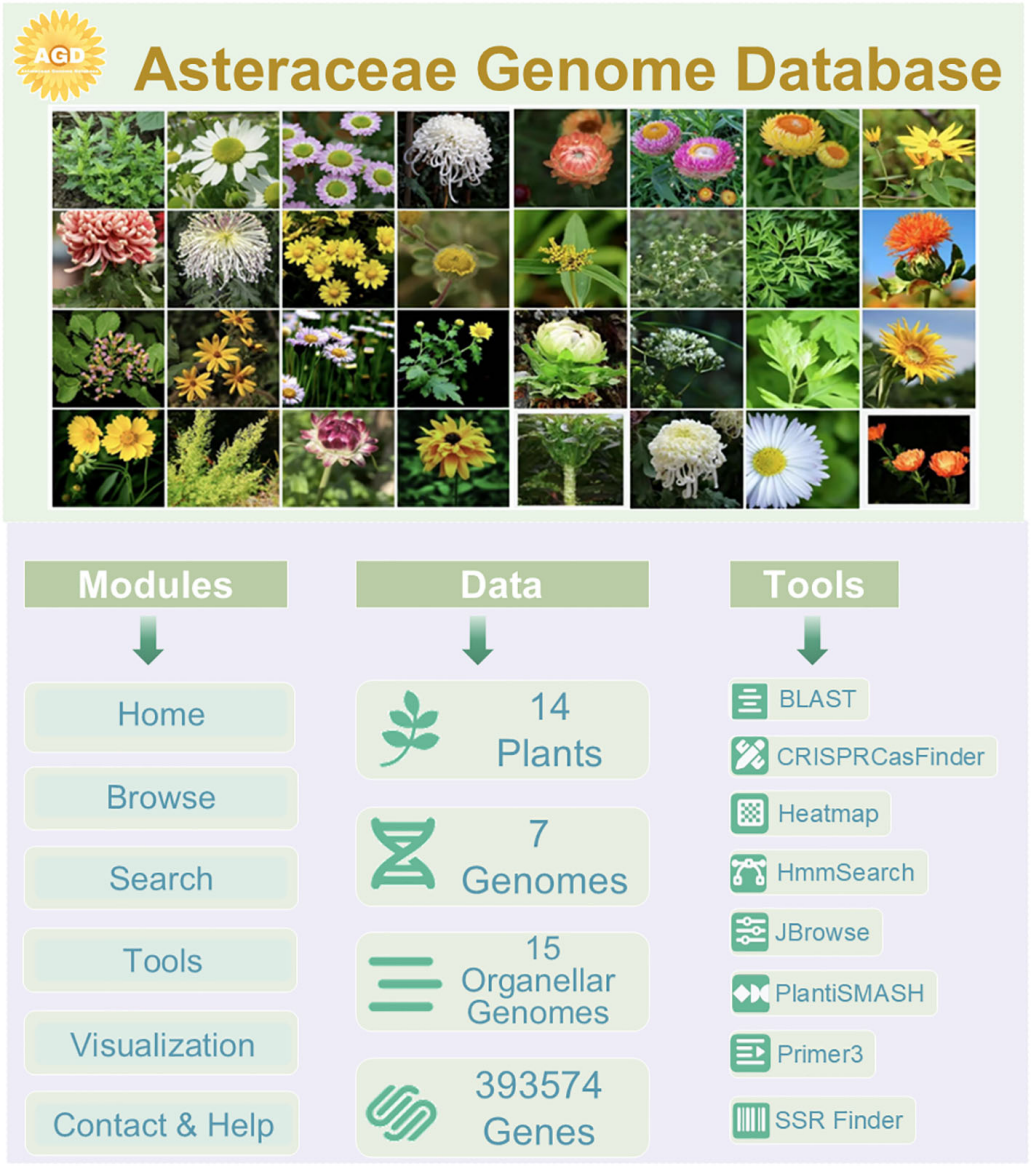
Framework of three parts at AGD.

AGD comprises three main parts, including modules, data, and tools (**Figure 2**). It incorporates six primary modules: Home, Browse, Search, Tools, Visualization, and Contact&Help, each serving distinct functions to facilitate user interaction and data exploration. We have collected genomic data from 40 Asteraceae species, of which seven genomic information that can be queried and downloaded, have been uploaded to the AGD. We are committed to continually improving and expanding the AGD. Furthermore, AGD includes organellar genomic data from 15 Asteraceae species, which adds valuable genetic information to the database. The database is further enriched with large of high-quality photographs showcasing a diverse array of Asteraceae plants.

AGD also integrates eight related tools with diverse functionalities and datasets. BLAST for ortholog recognition across a spectrum of plant species, SSR Finder for simple sequence repeats detection, and JBrowse for an immersive genome exploration experience. For protein domain identification, we have integrated HmmSearch, while primer design is facilitated through our proprietary tool. Furthermore, AGD now features PlantiSMASH for secondary metabolite analysis and CRISPRCasFinder for CRISPR-associated system identification, both of which have been embedded within the AGD for user convenience (**Figure 2**).

### 3.2 Browse

In the Browse module, users can browse through comprehensive list pages (plant, genome, organellar genomic); utilize interactive filters to narrow down datasets based on specific attributes, such as species hierarchy, assembly level, and herbal characteristics; and explore data subsets that possess the desired attribute. This module can also provide the detailed information, including herb names, habitats, genome version/level, data sources, characteristics, and descriptions.

### 3.3 Search

AGD has a separate search page where users can quickly find data of interest. The search box allows users to select a species or field and enter keywords. Recorded searches are displayed as a word cloud, and the results page provides a summary table with clickable hyperlinks for more details.

### 3.4 Tools

**Figure 3:**
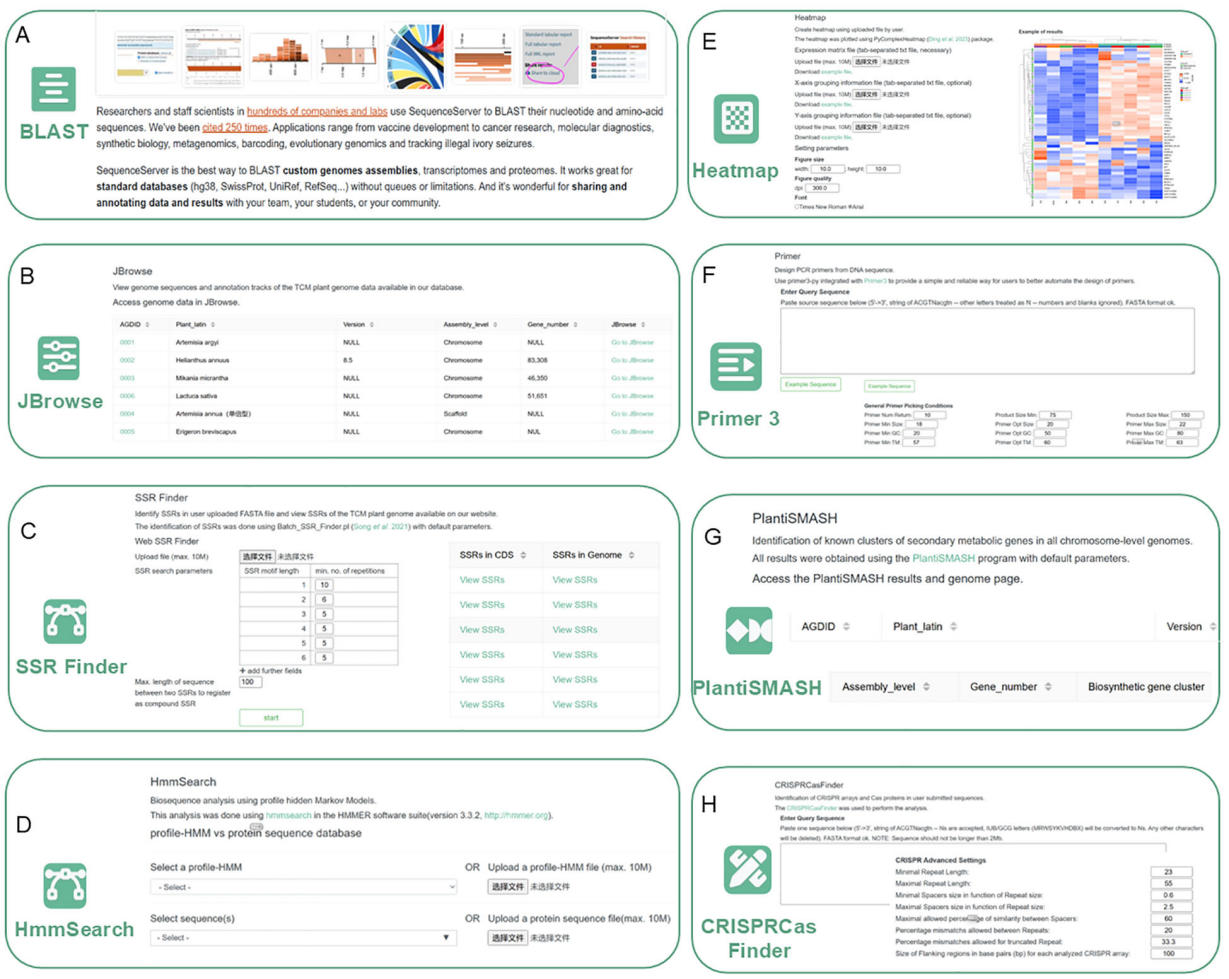
Eight tools at AGD. **(A)** Blast. **(B)** JBrowse. **(C)** SSR Finder. **(D)** HmmSearch. **(E)** Heatmap. **(F)** Primer3. **(G)** PlantiSMASH. **(H)** CRISPRCasFinder.

AGD has embedded several online analysis tools to facilitate the systematic analysis of Asteraceae plant genomes. For example, homology searches and the visualization of results can be performed by SequenceServer in BLAST. Users can input query sequences or upload a file in FASTA format, and select a database for the search. The available BLAST options are automatically set based on the query sequence type and selected database (**Figure 3A**). JBrowse can display the integrated data of three genomes and annotated genomic datasets. Users can upload their data for visualization and comparison with AGD datasets. JBrowse enables genome sequence browsing, viewing gene information, and data comparison (**Figure 3B**). In addition, the SSR Finder module identifies SSRs in uploaded sequences and displays SSRs found in AGD coding sequences (**Figure 3C**). HmmSearch analyzes gene families using profile-HMMs (**Figure 3D**) and Heatmap generates visual representations of data matrices (**Figure 3E**). Primer3 can be adopted to design primers for PCR experiments (**Figure 3F**), while PlantiSMASH predicts biosynthetic gene clusters in plants (**Figure 3G**) and CRISPRCasFinder identifies CRISPR-Cas systems in genomes (**Figure 3H**).

### 3.5 Visualization

We implement ECharts to display the data contained in AGD. Users can access this tool through the visualization buttons on the navigation bar, which serves as the starting point for exploring the database. The AGD visualization interface offers simple statistics, including the number of plants in the Asteraceae family and the number of Asteraceae and organellar genomes. Users can also examine detailed charts for specific taxonomic subsets by engaging with the corresponding category tabs. The taxonomic hierarchy of the flora is represented with a Sunburst diagram, which allows for the expansion of any segment upon user interaction, and is accompanied by a set of controls below the diagram to facilitate the retrieval of pertinent records. In the genomic data representation block, we include a donut chart featuring smoothed edges to delineate the distribution of genomes across various size spectra. Users can extract corresponding data entries by interacting with any segment of the chart.

### 3.6 Contact and help

We have included a feedback form within the contact module, tailored for users to conveniently submit their inquiries, concerns, and suggestions regarding various issues. Our email address is displayed on the contact page, ensuring swift and straightforward communication with our team. To strengthen the accessibility of the user interface, we present detailed step-by-step instructions on the help page on how to utilize the primary modules.

## 4 Discussion

From 2000 to 2020, 1,144 genomes of 782 plant species were sequenced (Xie et al., 2024). Compared to ~10 years ago, high-quality genome assembly has become relatively easier, and there has been a tremendous leap in genome assembly. Due to the remarkable advancements in sequencing technology, a vast array of species has been sequenced (Yang et al., 2024a), and a total of 2,836 genomes from 1,410 plant species was available by 2023 (Xie et al., 2024). Of course, the genome assembly quality has also improved rapidly (Yang et al., 2024b). These afforded the emergence of several databases dedicated to housing their genomes, such as the 1 K medicinal plant genome database (Su et al., 2022), the Rosaceae genome database (Jung et al., 2019), the cucurbit genomics database (Zheng et al., 2019), and the Portal of Juglandaceae (Guo et al., 2020), Traditional Chinese Medicine Plant Genome database Traditional Chinese Medicine Plant Genome database (TCMPG; http://cbcb.cdutcm.edu.cn/TCMPG/) (Meng et al., 2022), and so on (**Supplementary Table S1**). Asteraceae, the largest family of flowering plants, is renowned for its medicinal, horticultural, and ornamental value. However, research on these plants faces several challenges. The diverse habitats of the Asteraceae family have led to the widespread dispersion of its resources. Additionally, many Asteraceae species are polyploids with large and diverse genomes, posing significant challenges for scientific research due to their genetic complexity. Meanwhile, the continuous advancement of sequencing technologies has facilitated the extensive publication of genomic and genetic resources for various Asteraceae species.

The Global Compositae Database (https://www.compositae.org/gcd/index.php) boasts an extensive collection of approximately 33,057 recognized species. A large number of databases provide partial information on Asteraceae data, yet the data available is quite restricted, such as the GERDH databases, while offering valuable resources for horticultural crops, are limited in scope as they only cover a small number of closely related Asteraceae species (Cheng et al., 2023). According to the published plant genome website, 40 Asteraceae species have had their genomes sequenced, each with varying degrees of assembly completeness and distributed in different databases. Currently, genomes, organelle genomes, and some genetic resources of Asteraceae are distributed in different databases, resulting in the need to spend a lot of time collecting this information before many bioinformatics analyses, lacking a unique and comprehensive database that integrates a large amount of available information on Asteraceae genomics and genetic resources. We recognized that constructing an Asteraceae genome database provides researchers with a comprehensive and user-friendly genomics resource platform, which is very important for advancing Asteraceae genomics and promoting genetic breeding.

Based on this, the Asteraceae Genome Database (AGD) introduces 15 organelle genomes and 7 genomic information of Asteraceae that can be queried and downloaded, along with related genetic information, it provides a data update mechanism, improved user interface design, and advanced data analysis tools (including BLAST, JBrowse, SSR Finder, HmmSearch, Heatmap, Primer3, PlantiSMASH, and CRISPRCasFinder). As an integrated repository for genomic, genotypic, and taxonomic data, it is committed to promoting research on Asteraceae species.

In this work, we developed AGD to manage this wealth of data on the Asteraceae species effectively. It integrates genomic data from multiple species, offering a platform for comparative and functional genomics analysis. This integration is pivotal as it uncovers conserved and variable regions within the genomes, shedding light on gene functions and evolutionary patterns across the family. This strengthens phylogenetic studies, genetic breeding, and drug development specifically for Asteraceae plants. Moreover, we provide robust data analysis and visualization tools, as well as comprehensive and insightful data support for Asteraceae plant research, thereby propelling scientific advancements in related fields.

## 5 Conclusion

The AGD was established as an integrated database resource dedicated to collecting the genomic-related data of the Asteraceae family, including genomic datasets, organellar genomes, and phenotypic information. Equipped with a suite of useful tools, including BLAST, JBrowse, SSR Finder, HmmSearch, Heatmap, Primer3, PlantiSMASH, and CRISPRCasFinder, the AGD offers researchers valuable resources for genomic analysis. The database is freely accessible online at http://cbcb.cdutcm.edu.cn/AGD/. The AGD serves as a comprehensive repository of genome, genotype, and taxonomy data, and stands as a valuable resource for the entire research community of Asteraceae.

## Data Availability

The datasets presented in this study can be found in online repositories. The names of the repository/repositories and accession number(s) can be found in the article/**Supplementary Material**.
