# Supplementary material for: Asteraceae genome database: a comprehensive platform for Asteraceae genomics
Source: Front Plant Sci. 2024 Aug 19;15:1445365. doi: 10.3389/fpls.2024.1445365 (PMC11366637; doi:10.3389/fpls.2024.1445365)
Supplement: Supplementary file 1 [file Table1.docx]

Supplementary Material

## Supplementary Tables

Table S1 Summary of main genome databases for medicinal plants

| Species | Database | Links | References |
| --- | --- | --- | --- |
| Ginseng | Ginseng Genome Database | http://ginsengdb.snu.ac.kr / | (Jayakodi et al., 2018) |
| Orchid | Orchidstra 4.0 | http://orchidbase.itps.ncku.edu.tw/ | (Hsiao et al., 2021) |
| *Arctium lappa* | burdock database | http://210.22.121.250:41352/ | (Song et al., 2023) |
| Chrysanthemum | Chrysanthemum Genome Database | http://www.amwayabrc.com. | (Song et al., 2018) |
| *Ginkgo biloba* | GinkgoDB | https://ginkgo.zju.edu.cn/ | (Gu et al., 2022) |
| *Sacred lotus* | NGD | http://Nelumbo.biocloud.net | (Li et al., 2021) |
| Rosaceae | GDR | https://www.rosaceae.org/ | (Jung et al., 2019) |
| Cucurbit | CuGenDB | http://cucurbitgenomics.org | (Zheng et al., 2019) |
| Kiwifruit | KGD | <http://kiwifruitgenome.org/> | (Yue et al., 2020) |
| Sesame | SesameFG | http://ncgr.ac.cn/SesameFG/ | (Wei et al., 2017) |
| Holly | HollyGTD | <https://hollygdb.com/> | (Guo et al., 2023) |
| Juglandaceae | PJU | http://www.juglandaceae.net/ | (Guo et al., 2020) |
| *Brassica oleracea* | BoGDB | http://bogdb.com/ | (Wang et al., 2022) |
| Eschscholzia | Eschscholzia Genome DataBase | https://eschscholzia.kazusa.or.jp/ | (Hori et al., 2018) |
| Brassicaceae | TBGR | http://www.tbgr.org.cn | (Liu et al., 2022) |
| Amaranth | AGRDB | http://www.nbpgr.ernet.in:8080/AmaranthGRD/ | (Singh et al., 2023) |
| Passionfruit | PGD | http://passionfruit.com.cn. | (Yu et al., 2024) |
| Pitaya | PGMD | http://pitayagenomic.com/ | (Chen et al., 2022) |
| Cacao | Cacao Genome Database | https://www.cacaogenomedb.org/ | (Ficklin et al., 2011) |
| Malvaceae | MaGenDB | http://magen.whu.edu.cn/magendb/ | (Wang et al., 2020) |
| Euphorbiaceae | EupDB | http://eupdb.liu-lab.com/ | (Liu et al., 2024) |
| mulberry | MorusDB | http://morus.swu.edu.cn/morusdb/ | (Li et al., 2014) |
| *Cannabis Sativa* | CannabisGDB | [https://gdb.supercann.net](https://gdb.supercann.net/) | (Cai et al., 2021) |
| Citrus | Citrus Genome Database | https://www.citrusgenomedb.org/ | (Liu et al., 2022) |
| Brassicaceae | BRAD3.0 | http://www.brassicadb.cn/#/ | (Chen et al., 2022) |
| Mango | MangoBase | https://mangobase.org/easy_gdb/index.php | (Gómez-Ollé et al., 2023) |
| Carrot | CarrotDB | http://carrotdb.cn:7777/ | (Xu et al., 2014) |
| Ericaceae | TEGR | http://www.tegr.com.cn | (Wang et al., 2023) |
| *Ophiorrhiza pumila* | Ophiorrhiza pumila Genome DataBase | https://pumila.kazusa.or.jp/ | (Rai et al., 2021) |
| Solanaceae | SGN | https://solgenomics.sgn.cornell.edu/ | (Mueller et al., 2005) |

References

Cai, S., Zhang, Z., Huang, S., Bai, X., Huang, Z., Zhang, Y. J., et al. (2021). CannabisGDB: a comprehensive genomic database for Cannabis Sativa L. *Plant Biotechnol J* 19, 857–859. doi: 10.1111/pbi.13548

Chen, C., Li, F., Xie, F., Chen, J., Hua, Q., Chen, J., et al. (2022). Pitaya Genome and Multiomics Database (PGMD): A Comprehensive and Integrative Resource of Selenicereus undatus. *Genes (Basel)* 13, 745. doi: 10.3390/genes13050745

Chen, H., Wang, T., He, X., Cai, X., Lin, R., Liang, J., et al. (2022). BRAD V3.0: an upgraded Brassicaceae database. *Nucleic Acids Res* 50, D1432–D1441. doi: 10.1093/nar/gkab1057

Ficklin, S. P., Sanderson, L.-A., Cheng, C.-H., Staton, M. E., Lee, T., Cho, I.-H., et al. (2011). Tripal: a construction toolkit for online genome databases. *Database (Oxford)* 2011, bar044. doi: 10.1093/database/bar044

Gómez-Ollé, A., Bullones, A., Hormaza, J. I., Mueller, L. A., and Fernandez-Pozo, N. (2023). MangoBase: A Genomics Portal and Gene Expression Atlas for Mangifera indica. *Plants (Basel)* 12, 1273. doi: 10.3390/plants12061273

Gu, K.-J., Lin, C.-F., Wu, J.-J., and Zhao, Y.-P. (2022). GinkgoDB: an ecological genome database for the living fossil, Ginkgo biloba. *Database (Oxford)* 2022, baac046. doi: 10.1093/database/baac046

Guo, W., Chen, J., Li, J., Huang, J., Wang, Z., and Lim, K.-J. (2020). Portal of Juglandaceae: A comprehensive platform for Juglandaceae study. *Hortic Res* 7, 35. doi: 10.1038/s41438-020-0256-x

Guo, Z., Wei, J., Xu, Z., Lin, C., Peng, Y., Wang, Q., et al. (2023). HollyGTD: an integrated database for holly (Aquifoliaceae) genome and taxonomy. *Front Plant Sci* 14, 1220925. doi: 10.3389/fpls.2023.1220925

Hori, K., Yamada, Y., Purwanto, R., Minakuchi, Y., Toyoda, A., Hirakawa, H., et al. (2018). Mining of the Uncharacterized Cytochrome P450 Genes Involved in Alkaloid Biosynthesis in California Poppy Using a Draft Genome Sequence. *Plant and Cell Physiology* 59, 222–233. doi: 10.1093/pcp/pcx210

Hsiao, Y.-Y., Fu, C.-H., Ho, S.-Y., Li, C.-I., Chen, Y.-Y., Wu, W.-L., et al. (2021). OrchidBase 4.0: a database for orchid genomics and molecular biology. *BMC Plant Biol* 21, 371. doi: 10.1186/s12870-021-03140-0

Jayakodi, M., Choi, B.-S., Lee, S.-C., Kim, N.-H., Park, J. Y., Jang, W., et al. (2018). Ginseng Genome Database: an open-access platform for genomics of Panax ginseng. *BMC Plant Biol* 18, 62. doi: 10.1186/s12870-018-1282-9

Jung, S., Lee, T., Cheng, C.-H., Buble, K., Zheng, P., Yu, J., et al. (2019). 15 years of GDR: New data and functionality in the Genome Database for Rosaceae. *Nucleic Acids Res* 47, D1137–D1145. doi: 10.1093/nar/gky1000

Li, H., Yang, X., Zhang, Y., Gao, Z., Liang, Y., Chen, J., et al. (2021). Nelumbo genome database, an integrative resource for gene expression and variants of Nelumbo nucifera. *Sci Data* 8, 38. doi: 10.1038/s41597-021-00828-8

Li, T., Qi, X., Zeng, Q., Xiang, Z., and He, N. (2014). MorusDB: a resource for mulberry genomics and genome biology. *Database (Oxford)* 2014, bau054. doi: 10.1093/database/bau054

Liu, H., Wang, X., Liu, S., Huang, Y., Guo, Y.-X., Xie, W.-Z., et al. (2022). Citrus Pan-Genome to Breeding Database (CPBD): A comprehensive genome database for citrus breeding. *Mol Plant* 15, 1503–1505. doi: 10.1016/j.molp.2022.08.006

Liu, J., Li, Y., Li, J., Chen, W., Pan, B., Liu, A., et al. (2024). EupDB: An integrative and comprehensive functional genomics data hub for Euphorbiaceae plants. *Plant Commun* 5, 100683. doi: 10.1016/j.xplc.2023.100683

Liu, Z., Li, N., Yu, T., Wang, Z., Wang, J., Ren, J., et al. (2022). The Brassicaceae genome resource (TBGR): A comprehensive genome platform for Brassicaceae plants. *Plant Physiol* 190, 226–237. doi: 10.1093/plphys/kiac266

Mueller, L. A., Solow, T. H., Taylor, N., Skwarecki, B., Buels, R., Binns, J., et al. (2005). The SOL Genomics Network: a comparative resource for Solanaceae biology and beyond. *Plant Physiol* 138, 1310–1317. doi: 10.1104/pp.105.060707

Rai, A., Hirakawa, H., Nakabayashi, R., Kikuchi, S., Hayashi, K., Rai, M., et al. (2021). Chromosome-level genome assembly of Ophiorrhiza pumila reveals the evolution of camptothecin biosynthesis. *Nat Commun* 12, 405. doi: 10.1038/s41467-020-20508-2

Singh, A., Mahato, A. K., Maurya, A., Rajkumar, S., Singh, A. K., Bhardwaj, R., et al. (2023). Amaranth Genomic Resource Database: an integrated database resource of Amaranth genes and genomics. *Front Plant Sci* 14, 1203855. doi: 10.3389/fpls.2023.1203855

Song, C., Liu, Y., Song, A., Dong, G., Zhao, H., Sun, W., et al. (2018). The Chrysanthemum nankingense Genome Provides Insights into the Evolution and Diversification of Chrysanthemum Flowers and Medicinal Traits. *Mol Plant* 11, 1482–1491. doi: 10.1016/j.molp.2018.10.003

Song, Y., Yang, Y., Xu, L., Bian, C., Xing, Y., Xue, H., et al. (2023). The burdock database: a multi-omic database for Arctium lappa, a food and medicinal plant. *BMC Plant Biol* 23, 86. doi: 10.1186/s12870-023-04092-3

Wang, D., Fan, W., Guo, X., Wu, K., Zhou, S., Chen, Z., et al. (2020). MaGenDB: a functional genomics hub for Malvaceae plants. *Nucleic Acids Res* 48, D1076–D1084. doi: 10.1093/nar/gkz953

WANG Xiao-jing, W. Y. (n.d.). TEGR: A comprehensive Ericaceae Genome Resource database. *Journal of Integrative Agriculture*, 0. doi: 10.1016/j.jia.2023.11.026

Wang, Y., Ji, J., Fang, Z., Yang, L., Zhuang, M., Zhang, Y., et al. (2022). BoGDB: An integrative genomic database for Brassica oleracea L. *Front Plant Sci* 13, 852291. doi: 10.3389/fpls.2022.852291

Wei, X., Gong, H., Yu, J., Liu, P., Wang, L., Zhang, Y., et al. (2017). SesameFG: an integrated database for the functional genomics of sesame. *Sci Rep* 7, 2342. doi: 10.1038/s41598-017-02586-3

Xu, Z.-S., Tan, H.-W., Wang, F., Hou, X.-L., and Xiong, A.-S. (2014). CarrotDB: a genomic and transcriptomic database for carrot. *Database (Oxford)* 2014, bau096. doi: 10.1093/database/bau096

Yu, C., Wang, P., Zhang, S., Liu, J., Cheng, Y., Zhang, S., et al. (2024). Passionfruit Genomic Database (PGD): a comprehensive resource for passionfruit genomics. *BMC Genomics* 25, 157. doi: 10.1186/s12864-024-10069-9

Yue, J., Liu, J., Tang, W., Wu, Y. Q., Tang, X., Li, W., et al. (2020). Kiwifruit Genome Database (KGD): a comprehensive resource for kiwifruit genomics. *Hortic Res* 7, 117. doi: 10.1038/s41438-020-0338-9

Zheng, Y., Wu, S., Bai, Y., Sun, H., Jiao, C., Guo, S., et al. (2019). Cucurbit Genomics Database (CuGenDB): a central portal for comparative and functional genomics of cucurbit crops. *Nucleic Acids Res* 47, D1128–D1136. doi: 10.1093/nar/gky944
